# Co-occurrence of clozapine-related DRESS syndrome core clinical manifestations: results of a systematic review

**DOI:** 10.1192/j.eurpsy.2022.1854

**Published:** 2022-09-01

**Authors:** R. De Filippis, P. De Fazio, J. Kane, G. Schoretsanitis

**Affiliations:** 1 University Magna Graecia of Catanzaro, Department Of Health Sciences, Catanzaro, Italy; 2 The Zucker Hillside Hospital, Department Of Psychiatry, New York, United States of America; 3 University of Zurich, Psychiatric University Hospital, Zurich, Switzerland

**Keywords:** psychopharmacology, Clozapine-related DRESS syndrome, schizophrénia, drug hypersensitivity

## Abstract

**Introduction:**

Drug reaction with eosinophilia and systemic symptoms (DRESS) syndrome refers to a cluster of clinical symptoms/signs related to drug hypersensitivity. The main clinical features include fever, skin rash, eosinophilia, enlarged lymph nodes, atypical lymphocytosis, and involvement of at least one internal organ. Clozapine-related DRESS syndrome has been rarely reported, but this may be due to a different clinical presentation pattern compared to DRESS for other culprit drugs.

**Objectives:**

We aimed to assess clusters of main clinical features of clozapine-related DRESS.

**Methods:**

We ran a network analysis for clinical manifestations in the pooled sample of all previous published cases of clozapine-related DRESS.

**Results:**

We observed a triad of core symptoms (i.e., organ implication, fever, and eosinophilia) among DRESS criteria co-occurring in 59.3% (n=16) of 27 patients. The organs most likely to be involved in clozapine-related DRESS included lungs, liver, heart, and kidneys. Fever was also present in almost all cases (n=25 patients), while eosinophilia was observed in two thirds of the sample (n=18 patients).

**Conclusions:**

Regarding clinical manifestations clozapine-related DRESS may differ from DRESS for other culprit drugs as skin reaction is not very typical; thus, clinicians need to consider DRESS as a potential diagnosis even in absence of a skin reaction. When managing clozapine-treated patients with the core triad of organ implication, fever, and eosinophilia clinicians should consider guidelines for DRESS treatment.

**Disclosure:**

No significant relationships.

